# Latitudinal gradients and ocean fronts strongly influence protist communities in the southern Pacific Ocean

**DOI:** 10.1093/femsec/fiae137

**Published:** 2024-11-11

**Authors:** Daniela Sturm, Peter Morton, Gerald Langer, William M Balch, Glen Wheeler

**Affiliations:** The Marine Biological Association of the United Kingdom, The Laboratory, Citadel Hill, Plymouth, Devon PL1 2 PB, United Kingdom; School of Ocean and Earth Science, University of Southampton, Southampton SO14 3ZH, United Kingdom; National High Magnetic Field Laboratory, Tallahassee, FL 32310, United States; Institute of Environmental Science and Technology, Universitat Autònoma de Barcelona, Barcelona, 08193, Spain; Bigelow Laboratory for Ocean Sciences, 60 Bigelow Drive, East Boothbay, ME 04544, United States; The Marine Biological Association of the United Kingdom, The Laboratory, Citadel Hill, Plymouth, Devon PL1 2 PB, United Kingdom

**Keywords:** Pacific Ocean, protist, *Phaeocystis*, diversity, fronts

## Abstract

Protist communities in the southern Pacific Ocean make a major contribution to global biogeochemical cycling, but remain understudied due to their remote location. We therefore have limited understanding of how large-scale physical gradients (e.g. temperature) and mesoscale oceanographic features (e.g. fronts) influence microeukaryote diversity in this region. We performed a high-resolution examination of protist communities along a latitudinal transect (>3000 km) at 150°W in the central southern Pacific Ocean that encompassed major frontal regions, including the subtropical front (STF), the subantarctic front (SAF), and the polar front (PF). We identified distinct microbial communities along the transect that were primarily delineated by the positions of the STF and PF. Some taxa were not constricted by these environmental boundaries and were able to span frontal regions, such as the colonial haptophyte *Phaeocystis*. Our findings also support the presence of a latitudinal diversity gradient (LDG) of decreasing diversity of the protist community with increasing latitude, although some individual taxa, notably the diatoms, do not adhere to this rule. Our findings show that oceanographic features and large-scale physical gradients have important impacts on marine protist communities in the southern Pacific Ocean that are likely to strongly influence their response to future environmental change.

## Teaser

Protist communities in the southern Pacific Ocean are constrained by large-scale oceanographic fronts.

## Introduction

Microorganisms form the basis of the global food web and are essential for the functioning of marine ecosystems (Worden et al. 2015). Protists, a paraphyletic assemblage of diverse eukaryotic taxa, play a crucial role in nutrient and carbon cycling and serve as primary producers, consumers, and decomposers. Marine protists have been shown to be important indicators of environmental change, as they are sensitive to changes in temperature, salinity, and nutrient availability (Richardson and Schoeman 2004, Deppeler and Davidson 2017, Hutchins and Fu 2017). Monitoring protist populations through time therefore improves our understanding how marine ecosystems will respond to climate change, pollution, and other external stressors (Pawlowski et al. 2016, Stern et al. 2018). Detecting changes in protist communities will be impossible without a thorough grasp of current distribution and diversity patterns, particularly in remote oceanic regions like the Southern Ocean (SO), an important region for carbon sequestration, with over 40% of anthropogenically produced CO_2_-uptake occurring south of 30°S (Frölicher et al. 2015, Landschützer et al. 2015).

**Figure 1.**
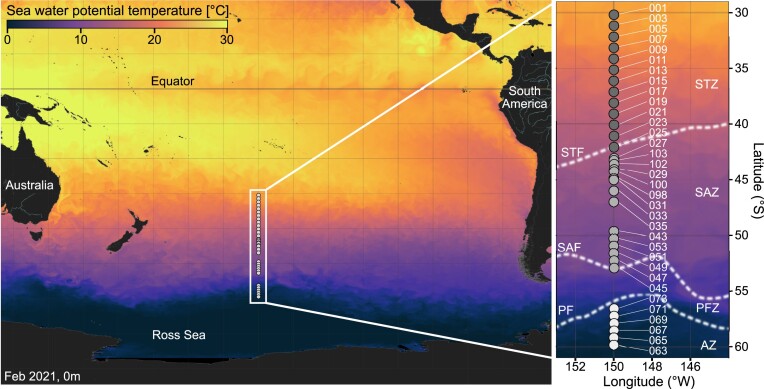
Sea surface temperature (°C) for February 2021 at 0 m depth. Insert shows study area and locations of the R/V Revelle #RR2004 stations. The different oceanographic fronts are indicated as follows: Subtropical Front (STF), Subantarctic Front (SAF), and Antarctic Polar Front (PF), according to Orsi et al. (1995). The areas between the fronts are referred to as Subtropical Zone (STZ), Subantarctic Zone (SAZ), Polar Frontal Zone (PFZ), and Antarctic Zone (AZ). We characterized three distinct environmental regions along the transect: an oligotrophic northern region (STZ), a temperate region, where macronutrients increase but silicate concentrations are low (SAZ + PFZ), and a polar region, where both macronutrients and silica concentrations are high (AZ). Temperature data from Copernicus Marine Service.

The central southern Pacific Ocean (140°W–180°W) constitutes a unique environment for studying marine protist communities due to its remote and mostly pristine nature. Its geography allows the study of protist distribution across thousands of kilometres, relatively unencumbered by even small land masses. However, adverse weather conditions and its vastness, stretching about 10 000 km between Chile and Australia, make it a particularly difficult region to study. The marine environment of the southern Pacific Ocean is influenced by complex oceanographic conditions, which include circumpolar fronts, upwelling zones, and large-scale ocean circulation patterns (Orsi et al. 1995). Generally, the SO is characterized by a large latitudinal temperature gradient and very distinct oceanic regions with different nutrient regimes, separated by oceanic fronts (Fig. 1). There is still a need to understand the contribution of large-scale gradients and distinct oceanographic features to microbial diversity in this region.

Two primary metrics for assessing large-scale biodiversity trends in terrestrial environments are the latitudinal diversity gradient (LDG) and Rapoport’s rule (Mittelbach et al. 2007, Jablonski et al. 2017). The LDG refers to the pattern of increasing biodiversity from the poles towards the equator, with highest species richness typically found in tropical regions. Several hypotheses have been proposed to explain this trend, such as the relationship between temperature and productivity or increased stability in environmental conditions at low latitudes (Mittelbach et al. 2007, Jablonski et al. 2017). In contrast to terrestrial systems, there are few direct measurements of large-scale biogeographical biodiversity patterns in marine microorganisms. Molecular data from the *Tara Oceans* expedition indicated the LDG may also apply to marine microbial communities, with a decrease in marine protist diversity at higher latitudes driven by decreasing ocean temperatures (Ibarbalz et al. 2019). Studies using a global ecosystem model also observed a decrease in phytoplankton diversity at high latitudes, which was attributed to greater seasonal variability in these regions (Barton et al. 2010). Rapoport’s rule states that the latitudinal ranges of species tend to be greater at higher latitudes than lower latitudes. This has been confirmed for marine bacterioplankton (Amend et al. 2013, Sul et al. 2013), while others concluded that this pattern only applies locally in animals and plants generally (Rohde 1996, Gaston et al. 1998). However, a study in the SO found contrasting evidence, showing decreasing latitudinal range with increasing distance from the equator for archaea, bacteria, and eukaryotic microbes (Raes et al. 2018). Greater spatial and temporal resolution of microbial biodiversity data across large-scale gradients is required to resolve these conflicting issues and determine whether both the LDG and Rapoport’s rule hold true for marine protists.

Oceanographic features also have the potential to influence protist communities within SO (Wolf et al. 2013, Raes et al. 2018, Gutiérrez-Rodríguez et al. 2022). The Subantarctic zone (SAZ) is the largest marine high-nutrient, low-chlorophyll (HNLC) province, where phytoplankton productivity is generally limited by bottom-up (iron, light) and top-down (grazing) factors (Hiscock et al. 2003, Doblin et al. 2011). It includes three important frontal regions, the subtropical front (STF), the subantarctic front (SAF), and the polar front (PF), with waters between the SAF and the PF referred to as the polar frontal zone (PFZ) (Fig. 1) (Orsi et al. 1995). Based on traditional methods enumerating protist communities using light microscopy and pigment analysis, the SAZ has been established as an important transitionary boundary between the dominance of coccolithophores that construct calcium carbonate shells in the north (the ‘Great Calcite Belt’) and diatoms with silica frustules in the south (Trull et al. 2001, Honjo 2004, Balch et al. 2016). South of the PF, cold (<2°C) Antarctic waters make up the Antarctic Zone (AZ). Here, biological production undergoes strong seasonality and mixed-layer deepening during winter storms brings nutrient-rich water to the surface fuelling spring phytoplankton blooms, predominantly made up of heavily silicified diatoms (Abbott et al. 2000, Rigual-Hernández et al. 2015).

Molecular techniques have been used extensively to study and monitor protist biodiversity. Metabarcoding using common marker genes such as 18S rRNA gene has enabled researchers to establish novel biogeographic patterns, discover novel lineages without cultured representatives, and identify much greater diversity than previously thought (Santoferrara et al. 2020). Metabarcoding enables the efficient analysis of a substantial quantity of samples, offering both time and cost effectiveness, coupled with high sensitivity and high taxonomic resolution. However, care needs to be taken with interpretation of metabarcoding datasets, as results can vary substantially depending on a range of technical and biological factors. For instance, use of the V9 region of the 18S rRNA gene has unveiled the considerable diversity and abundance of diplonemid flagellates, contrasting with their limited detection using the more commonly used V4 region (Flegontova et al. 2016). Other marker genes might offer improved resolution for species within specific taxa, such as 28S rRNA gene for haptophytes, although reference databases for non-18S regions are limited (Gran-Stadniczeñko et al. 2019). Another major source of bias in DNA metabarcoding is variability in gene copy number. Copy numbers for 18S rRNA gene can vary substantially between protist lineages, leading to substantial overestimation of abundance in some lineages and underestimation of others (Gong and Marchetti 2019, Martin et al. 2022). DNA metabarcoding is therefore able to provide unique insight into the composition of protist communities, but these considerations must be carefully applied in the interpretation of these datasets.

In this study, we used high-throughput next-generation sequencing with 18S rRNA gene metabarcoding to assess protist community structure and diversity along a 30-degree meridional transect in the remote southern Pacific Ocean along 150°W. We aimed to determine whether protist community structure and diversity differed with latitudinal gradients and examine whether protist communities were also constrained by oceanographic fronts. Our results indicate that both of these characteristics strongly influence protist communities in the southern Pacific Ocean and have implications understanding food webs, productivity, and carbon cycling in transitional ocean environments.

## Materials and methods

### Study area and sample collection

Samples for this study were taken onboard the *R/V Roger Revelle* (cruise #RR2004) in the Southern Pacific Ocean (Fig. 1) between 07 January 2021 and 08 February 2021 along a meridional transect at 150°W from 30°S to 60°S (∼3333 km).

Thirty-four stations were visited, and two samples were taken at each station, one from the surface (SA; sampled from the uppermost Niskin bottle at depths between 5 and 13 m) and one from the deep-chlorophyll fluorescence maximum (DCM; determined by CTD downcast as maximum chlorophyll fluorescence at depths between 13 and 150 m) in Niskin bottles mounted on a CTD-rosette. Samples of 1–2 l of water were collected in acid-cleaned Teflon FEB bottles (1.5% HCl), which were rinsed three times with Milli-Q water and subsequently rinsed and filled with sampling seawater at the respective station and depth. Samples were filtered through 0.22 µm 47 mm diameter mixed cellulose ester filters (FisherSci, USA) at 0.5-1 bar (Gast pump, p/n DOA-P704-AA), using a custom vacuum filtration rig with a Teflon 100-ml reservoir and support grid. The exact filtration amount was dependent on local biomass to avoid clogging of the filters. No prefiltration was carried out, as the abundance of metazoans was negligible. Filters were folded with ethanol-cleaned forceps and placed in acid-washed (10% HCl) 1.5 ml microcentrifuge tubes (FisherSci, p/n 05–408–129). Samples were preserved in RNAlater (Invitrogen, USA) and stored at −80°C.

### Hydrographic conditions and Nutrients

Temperature, salinity, and chlorophyll *a* fluorescence were measured using a Seabird SBE 9 CTD and WETStar fluoromoeter (Sea-Bird Scientific, USA), mounted on the rosette containing 24 Niskin bottles. Measurements of dissolved inorganic nutrients (nitrate, nitrite, ammonium, phosphate, silicate) and dissolved oxygen analysed were carried out onboard by the Scripps Oceanographic Data Facility (ODF) group according to the methods used by Atlas et al. 1971, Hager et al. 1972, and Gordon et al. 1993. For detailed methods, see Scripps Chemistry Services: https://scripps.ucsd.edu/ships/shipboard-technical-support/odf/chemistry-services. Values for nitrate, nitrite, and ammonium were combined for total dissolved inorganic nitrogen (DIN).

### DNA extraction, amplification, and sequencing of rRNA fragments

Filters for DNA analysis were processed with the ZymoBIOMICS DNA Miniprep Kit (Zymo Research, USA) according to the manufacturer’s instructions. Purity and quality of the extracted DNA were assessed with Nanodrop. Library preparation and sequencing were conducted by the NU-OMICS facility at Northumbria University, UK (https://earthmicrobiome.org/protocols-and-standards/18s/). Samples were PCR-amplified using the universal eukaryotic primers Illumina_Euk_1391f and Illumina_EukBr (Amaral-Zettler et al. 2009) targeting the hypervariable V9 region of the nuclear gene that encodes 18S rRNA gene (Supplementary Table S1). The following conditions were used: 94°C for 3 min, then 35 cycles of 94°C 45 s, 57°C 60 s, and 72°C 90 s, followed by 72°C for 10 min. The V9 18S region was chosen for this study as it was used by other large-scale metabarcoding studies such as the *Tara Oceans* expedition, enabling us to contextualize our data globally (Von Dassow et al. 2015). The V9 region was further found to demonstrate improved accuracy of eukaryotic phytoplankton community composition (Bradley et al. 2016), as well as being less prone to generating PCR biases and allowing the discrimination of taxa over a greater phylogenetic depth compared to other commonly used marker regions such as V4 (De Vargas et al. 2015, Catlett et al. 2020). Sequencing was conducted on an Illumina MiSeq using 250 + 250 bp paired-end V3 chemistry. Sequence and metadata are accessible through the NCBI Sequence Read Archive (SRA) under accession number PRJNA1044770.

### Sequence processing

Raw reads were preprocessed using ‘Cutadapt’ to remove adapter sequences, primers, and other types of unwanted sequences (Martin 2011). We processed paired-end reads according to the ‘DADA2’ pipeline (Callahan et al. 2016) in R-3.5.1. Reads were truncated and trimmed to remove low-quality reads and primers, respectively. At most 2 (forward reads) and 3 (reverse reads) errors were allowed during filtering. Settings of ‘filterAndTrim’ were set to truncLen=c130,120, maxN=0, maxEE=c(2,3), truncQ=2, rm.phix=TRUE. After dereplicating forward and reverse reads, amplicon sequence variants (ASVs) were resolved at single nucleotide resolution. Compared to traditional operational taxonomic units (OTUs), this method provides greater accuracy and reproducibility (Callahan et al. 2016). ASVs may not accurately correspond to distinct species, thus warranting caution when comparing metabarcoding data to traditionally defined species. Subsequently, paired-end reads were merged, and chimaeras identified and removed using the ‘removeBimeraDevono’ function of DADA2 in R. Approximately 2% of merged reads were identified as chimeric. The taxonomy of ASVs was assigned in DADA2 using ‘assignTaxonomy’ with the naive Bayesian classifier method and the PR^2^ database from Guillou et al. 2012 modified by De Vargas et al. 2015 to refine taxonomic assignments to the V9 18S region (V9_PR^2^ database W2 from De Vargas et al. 2015) (http://taraoceans.sb-roscoff.fr/EukDiv/#pr2). Read numbers per sample ranged from 26 444 to 194 511 with a mean of 53 653 reads. Sequence reads were randomly subsampled to an even depth of 26 444 reads per sample. ASVs classified as Archaea, Bacteria, Metazoa, Streptophyta, and Fungi were removed from the dataset before subsequent analysis of protist community. Roughly 15% of ASVs were unassigned at taxonomic ranks ‘below’ Kingdom, i.e. were classified as unknown Eukaryotes (∼37% at Phylum level). As many of these ASVs may represent clades for which no corresponding sequence is available, they were not removed from the dataset. Data were transformed to relative abundances using the ‘transform: compositional’ function within the ‘microbiome’ package (Version 1.10.0). For ordination and IndVal analyses, rare ASVs were pruned prior to transformation to avoid skewing of major trends. ASVs were considered as rare when they occurred <3 times in <10% of samples. R scripts used for DADA2 sequence processing and Phyloseq assignments can be found at https://github.com/divedaniela/FEMS_MicrobialEcology_Sturmetal2024.

### Analysis of protist biogeography

Frontal regions in the Southern Pacific, particularly the STF, can exhibit considerable seasonal, interannual, and decadal spatial variability (Kim and Orsi 2014, Behrens et al. 2021). Due to this variability, we used *in situ* observations of surface nutrient concentrations to define three distinct environmental regions. (1) The oligotrophic northern region, representing the STZ, was defined by low levels of dissolved inorganic nitrogen (DIN) (<1 µmol ;^−1^). (2) The temperate region, which included the SAZ and PFZ, was defined by higher concentrations of DIN (>2 µmol l^−1^), but low concentrations of silicate (<5 µmol l^−1^). (3) The polar region, which included the AZ, was defined by higher concentrations of silicate (>10 µmol l^−1^). The transition between the polar and temperate regions agreed closely with multi-year mean position of the PF (which shows little interannual variability at 150°W) (Kim and Orsi 2014). While the STF exhibits much greater seasonal and interannual variability, *in situ* measurements of temperature at 100 m depth (Oliver et al. 2023) indicated that the position of the STF corresponded closely to the transition between the temperate and oligotrophic regions (Orsi et al. 1995, Behrens et al. 2021).

Statistical analysis was carried out in R and metabarcoding data was explored using R package ‘phyloseq’ (McMurdie and Holmes 2013). Figures were made with ‘ggplot2’ (Wickham et al. 2016). To determine the overall and phylum-specific LDG, we analysed the alpha community metrics ASV richness and Shannon’s diversity Index (H’) along the transect using the ‘estimate_richness’ function within the ‘phyloseq’ package. To calculate Rapoport’s rule using Steven’s method, data were filtered to exclude ASVs that only occurred at one station along the transect (Stevens 1992). Surface and DCM values were averaged to obtain a combined presence/absence value for each latitude. Absolute latitudinal ranges of ASVs were calculated as the difference between the maximum and minimum latitudes where an ASV was present and averaged for each sample.

Assemblage-wide variation in protist community structure within the surveyed area was determined via ordination by non-metric multidimensional scaling (NMDS) based on Bray–Curtis dissimilarity coefficients. Vectors for environmental variables were plotted using the function ‘envfit’. To determine whether the centroids of the NMDS clusters are significantly different, we computed permutational multivariate analysis of variance (PERMANOVA) based on the Bray–Curtis distance matrix (Anderson 2001). We used analysis of similarity (ANOSIM) between the three factors to confirm that distances between groups are greater than within groups.

To determine shifts in trophic mode along the transect, we selected a sub-set of the 20 most abundant phyla based on their associated ASV abundances, and categorized these phyla into their respective primary or predominant trophic strategies based on information available in the ‘World Register for Marine Species’ (WoRMS) and literature review of specific taxa (Supplementary Table S2). We then summarized the relative abundances of each taxonomic group to obtain the proportion of each trophic strategy at all stations and their inherent meander locations. This analysis was aimed at examining broad shifts in community function, although it should be considered that not all organisms within each taxonomic group may utilize this trophic mode. We also did not attempt to resolve more complex heterotrophic feeding modes, such as parasitism, which is common in the Syndiniales. Marine protists can exhibit distinct feeding modes such as mixotrophy, parasitism, or osmotrophy, although predicting trophic mode at this higher resolution remains challenging for organisms that are not well-characterized. Future efforts to distinguish these trophic modes will provide greater insight into the function of marine microbial communities.

To identify which clades contribute most to differences in protist assemblage within the three regions as well as different depths, we used indicator value analysis (IndVal, Dufrêne and Legendre 1997). IndVal assesses the degree of fidelity (frequency of occurrence) and specificity (mean abundance within a cluster compared to the other clusters) of a species to a given factor and is therefore superior to analysis of just abundance.

Diversity indices, ASV abundances, and trophic strategy abundances were correlated against environmental parameters using Spearman’s Rank correlations using function cor.test from the ‘stats’ R package (Version 4.0.3) and visualized using corrplot from the ‘corrplot’ package (Version 0.90). Diversity relationships were assessed with linear regressions. Correlation results for individual clades were reported if the correlation coefficient R > 0.40 and *P* < 0.05.

For the dotplot analysis, we examined the 30 most abundant ASVs along the transect. To ensure meaningful comparisons and avoid obscuring lower abundance ASVs, we employed z-score transformations on their relative abundances. The z-score transformation, implemented using the ztransform function from the microbiome package, standardizes the data by centring it around the mean and scaling it by the standard deviation. This normalization facilitates a consistent scale for comparing ASVs, enabling the identification of patterns across the transect. Taxonomic identities of these ASVs were investigated in more detail using the National Center for Biotechnology Innovation (NCBI) BLASTn function (https://blast.ncbi.nlm.nih.gov/Blast.cgi). The dendogram was added with hierarchical cluster analysis using the R function hclust from the stats package using average linkage to measure the distance between clusters (https://www.rdocumentation.org/packages/stats/versions/3.6.2/topics/hclust).

## Results

### The transect encompasses three distinct environmental regions

**Figure 2.**
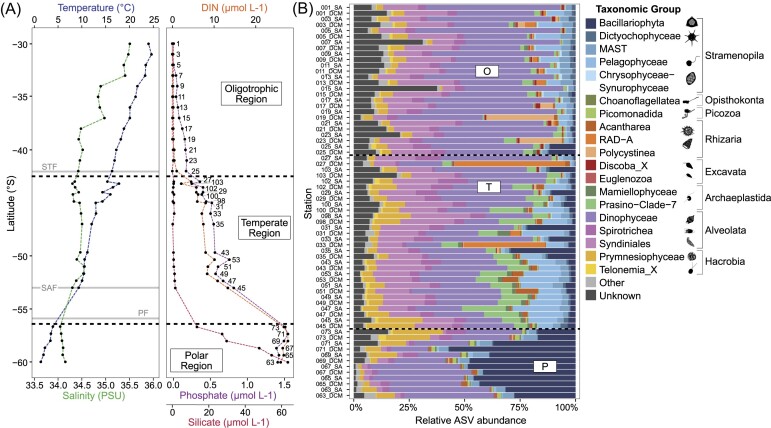
Environmental conditions and community composition along the transect. (A) Surface environmental conditions along the transect and throughout the defined regions. Left panel: Temperature and salinity from north to south. Solid lines represent the frontal boundaries encountered along the transect, and dotted lines represent the boundaries of the defined environmental regions. Right panel: Increasing dissolved inorganic nitrogen (DIN), phosphate, and silicate. From north to south. (B) Relative abundance of the 20 most abundant protist taxonomic groups (as assigned by V9_PR^2^ database W2) along the transect at the surface (SA) and deep chlorophyll maximum (DCM) as determined by 18S rRNA gene metabarcoding.

The transect crossed three major frontal systems: the Subtropical Front (STF), the Subantarctic Front (SAF), and the PF (Fig. 1). This resulted in three distinct environmental regions (defined in the section ‘Materials and methods’) along the transect that reflect the distinct oceanographic zones: (1) the oligotrophic northern region (representing the STZ), where nutrients were very low; (2) the temperate region, which included the SAZ and PFZ, where nutrients were higher but silicate remained low; and (3) the polar region, representing the AZ, characterized by higher concentrations of silicate (Fig. 2A).

The mean surface water temperature of the oligotrophic region was 20.5 ± 2.8°C, decreasing to 12.9 ± 2.7°C in the temperate region and 2.7 ± 1.0°C in polar region. Salinity also exhibited a southward decrease, from 34.9 ± 0.4 psu in the oligotrophic region to 34.1 ± 0.0 psu in the polar region. DIN concentrations increased from 0.15 ± 0.3 µmol l^−1^ (0.11 ± 0.08 µmol l^−1^) in the oligotrophic region to 26.9 ± 0.7 µmol l^−1^ (1.7 ± 0.07 µmol l^−1^) in the polar region with all regions being significantly different (all *P* values < 0.001). Silicate concentrations increased steadily south of the PF (mean 39.4 ± 18.9 µmol l^−1^ in the polar region), with very low silicate concentrations in the oligotrophic and the temperate regions (0.4 ± 0.2 µmol l^−1^ and 0.8 ± 0.4 µmol l^−1^, respectively). Surface chlorophyll fluorescence increased from 0.15 ± 0.4 µg l^−1^ in the oligotrophic region to 0.95 ± 0.6 µg l^−1^ in the polar region (*P* < 0.01).

### Overall community composition

After quality control, filtering, and normalization of sequences, 6266 ASVs were assigned for the 68 sampling locations along the transect with a maximum of 851 ASVs at Station 29_SA and a minimum of 193 ASVs at Station 69_SA. ASVs were assigned into 11 Supergroups, 24 Divisions, and 72 Phyla. The largest portion of ASVs was assigned to the Alveolate supergroup, mainly the flagellated mixotrophic dinoflagellates (Dinophyceae), and heterotrophic Syndiniales and Spirotrichea (Fig. 2B and Supplementary Table S3). Alveolates have high copy numbers for the 18S rRNA gene, resulting in a high relative abundance in metabarcoding libraries (Martin et al. 2022). Stramenopile ASVs were also abundant, including the silica-encased photosynthetic diatoms (Bacillariophyta), the photosynthetic Pelagophyceae, and the heterotrophic uncultured marine stramenopiles (MAST). Other clades that made up a substantial proportion of ASVs were the photosynthetic Prymnesiophyceae (Hacrobia), which include the calcifying coccolithophores, and chlorophyte Prasino-Clade-7 (Archaeplastida). Rhizaria ASVs had a lower relative abundance, but still constituted a substantial part of ASVs, with silicified radiolarians RAD-A, Polycystinea, and Acantharea all prominent (Fig. 2B).

### Distinct protist community composition between regions

**Figure 3.**
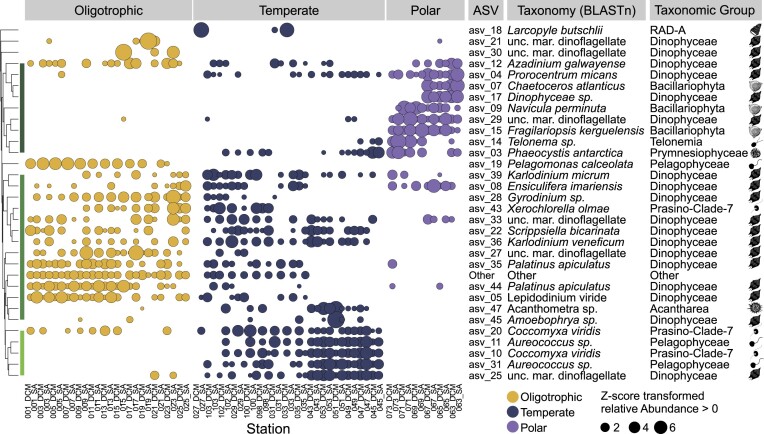
Trends in ASV distribution along the transect. Dot plot representation of relative, z-transformed abundance counts of the most abundant 30 ASVs presenting only abundances that positively deviate from the mean (z-score cutoff > 0). We identified three broad clusters within the different environmental regions: ASVs which are predominantly abundant in the polar region, ASVs which are predominantly abundant within the oligotrophic but also present in the temperate region, and ASVs which are predominantly abundant in the temperate region and absent in the polar region. Dendogram clusters ASVs with similar abundance patterns using average linkage.

To determine whether community composition changed between regions, we first examined the distribution of the 30 most abundant ASVs (Fig. 3). Hierarchical cluster analysis revealed three distinct clusters of ASVs with differing distributions across the three environmental regions. Cluster 1 consisted of ASVs that were primarily abundant in the polar region and included several diatom and dinoflagellate taxa. Cluster 2 consisted of ASVs that were primarily abundant in the oligotrophic region, although many were also present in the temperate region. This cluster consists primarily of dinoflagellate ASVs. Cluster 3 comprised of ASVs that were most abundant in the temperate region and consisted primarily of flagellates such as the Prasino-Clade-7 (Chlorophyta) and the Pelagophyceae (Stramenopiles).

**Figure 4.**
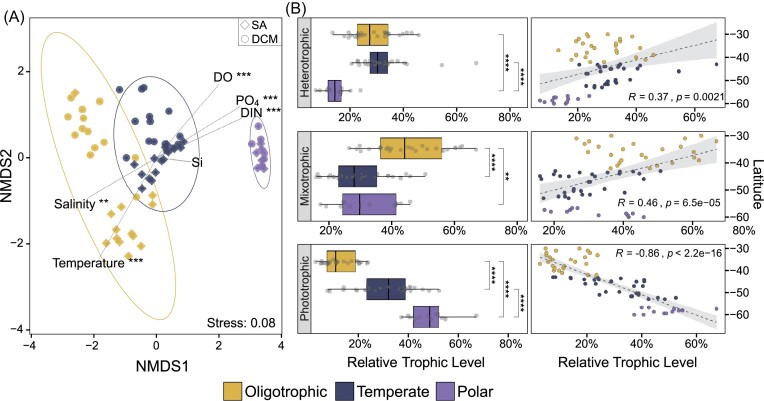
Community structure and trophic strategies along the transect. (A) Non-metric multidimensional scaling (NMDS) of Bray–Curtis dissimilarities in the oligotrophic, temperate, and polar regions. Ellipses indicate 95% confidence. Vector length and direction correspond to relative size effect and correlation to each axis, respectively. The shape of the points represents the sampling depth (SA: diamond, DCM: circle). (B) Relative abundance of trophic strategies in the three different regions (left) and relative abundance of trophic strategies with latitude. *P* < 0.0001 ^****^, *P* < 0.001 ***, *P* < 0.01 **, *P* < 0.05 *.

Ordination analysis based on Bray–Curtis dissimilarity indices (Fig. 4A) revealed that the three sampling regions were significantly different in their community structure (ANOSIM, R = 0.793, *P* = 0.001). The permutation test (envfit) showed that the environmental variables relevant for explaining this pattern were temperature (*r*  ^2^ = 0.26, *P* = 0.001), phosphate (*r*  ^2^ = 0.25, *P* = 0.001), DIN (*r*  ^2^ = 0.24, *P* = 0.001), dissolved Oxygen (*r*  ^2^ = 0.20, *P* = 0.001), and salinity (*r*  ^2^ = 0.17, *P* = 0.004). Silicate only constitutes a significant variable when comparing the temperate to the polar region (*r*  ^2^ = 0.27, *P* = 0.001). Stations in the polar region clustered tightly together, i.e. were highly similar in terms of their community structure, whereas stations in the oligotrophic region exhibited greater variability. This variation appears to be primarily driven by differences between the surface (SA) and deep chlorophyll maximum (DCM) communities.

We investigated whether these changes in community composition were reflected in distinct trophic strategies between the regions by comparing relative changes in the proportion of hetero-, mixo-, and phototrophic groups. We found that ASVs belonging to taxa with heterotrophic and mixotrophic feeding strategies exhibited greater relative abundance in the oligotrophic and temperate regions and were positively correlated with decreasing latitude (Fig. 4B). Phototrophic taxa were least abundant in the oligotrophic region and most abundant in the polar region, as well as strongly positively correlated with increasing latitudes. It is important to note that these analyses are based on ASV relative abundance rather than cell number or biomass, so the lower relative abundance of alveolate ASVs in polar waters may be driving these trends. We further found that relative abundances of heterotrophic and mixotrophic taxa were not constrained across the boundary of the oligotrophic and temperate regions and the temperate and polar regions, respectively (Fig. 4B).

### Indicator phyla for distinct protist communities in the southern Pacific Ocean

To examine the composition of the different communities within each region in greater detail, we used Indicator Value (IndVal) analysis to determine phyla that act as indicators of each region (Table 1). The strongest indicator phylum for the oligotrophic region was Discoba_X. The Discoba are a group of unicellular protists that includes *Euglena* and *Diplonema*, as well as parasites such as trypanosomes (Burki et al. 2020). Other indicator phyla for the oligotrophic region included Chrysophyceae-Synurophyceae and the photosynthetic Dictyochophyceae, which produce an intrinsic siliceous skeleton in one stage of their life cycle. These two phyla belong to the stramenopiles. Dinoflagellates are another indicator clade for the oligotrophic region, with decreasing abundance from north to south (Supplementary Fig. S1).

**Table 1.** Indicator taxa for the oligotrophic, temperate, and polar regions (as well as combinations) for the most abundant 25 taxa along the transect.

Region	Taxonomic group 1	Taxonomic group 2	IndVal stat	*P* value
**Oligotrophic**	Discoba_X	Excavata	0.66	0.001***
	Chrysophyceae-Synurophyceae	Stramenopila	0.57	0.001***
	Dinophyceae	Alveolata	0.53	0.001***
	Dictyochophyceae	Stramenopila	0.46	0.002**
	Polycystinea	Rhizaria	0.27	0.036*
**Temperate**	Prasino-Clade-7	Archaeplastida	0.77	0.001***
	Pelagophyceae	Stramenopila	0.55	0.001***
	Syndiniales	Alveolata	0.51	0.001***
	Mamiellophyceae	Archaeplastida	0.50	0.001***
	Acantharea	Rhizaria	0.33	0.017*
**Polar**	Bacillariophyta	Stramenopila	0.89	0.001***
	Picomonadida	Picozoa	0.74	0.001***
	Choanoflagellatea	Opisthokonta	0.65	0.001***
	Euglenozoa	Excavata	0.43	0.002*
	MAST	Stramenopila	0.33	0.014*
**Oligotrophic + Temperate**	Syndiniales	Alveolata	0.85	0.001***
	Dictyochophyceae	Stramenopila	0.57	0.001***
**Temperate + Polar**	Prymnesiophyceae	Hacrobia	0.32	0.031*

The chlorophytes Prasino-Clade-7 (IndVal value 0.77) and Mamiellophyceae (0.50) are indicator phyla of the temperate region, along with the Pelagophyceae (stramenopiles) (0.55), which are abundant overall in the temperate region and virtually absent in the polar region, but also found at the DCM in the oligotrophic region. The rhizarian Acantharia further shows a distinct peak in abundance at the south end of the temperate region, although its indicator value (0.33) is not as high as others.

The strongest indicator phyla in the polar region are the diatoms, which are virtually absent in the oligotrophic region and only show occasional spikes of abundance along the temperate region. Other strong indicator phyla for the polar region the choanoflagellates and the picomonads. Both these groups increased in abundance towards the southern end of the temperate region and are abundant in the polar region (Supplementary Fig. S1).

### Large-scale trends in protist diversity along the transect: Rapoport’s rule and the LDG

**Figure 5.**
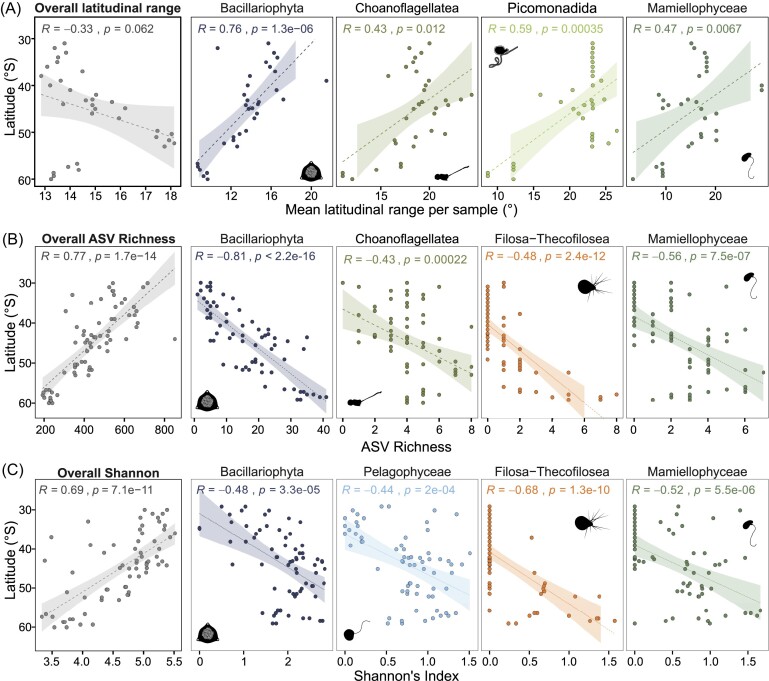
Linear regression (Spearman) of diversity along the transect for the entire protist community, as well as clades that deviate from the overall trend. (A) No relationship between mean latitudinal range per sample and latitude (Rapoport’s rule) in the overall protist community. Most protist clades exhibit increasing latitudinal range towards the pole, with some exceptions; the latitudinal range of Bacillariophyta, Choanoflagellatea, Picomonadida, and Mammiellophyceae decreases towards the pole. (B) Decreasing ASV Richness in the overall protist community polewards, and increasing richness polewards in the Bacillariophyta, Choanoflagellatea, Filosa-Thecofilosea, and Mammiellophyceae. (C) Decreasing Shannon’s Index polewards in the overall protist community, increasing Shannon's Index polewards in the Bacillariophyta, Pelagophyceae, Filosa-Thecofilosea, and Mammiellophyceae. Note the different x-axis scales.

The substantial physical gradients across the transect may result in broader scale effects on biodiversity and latitudinal range, as identified by the LDG and Rapoport’s rule. We did not find a significant trend in latitudinal range with increasing latitude (correlation coefficient R = −0.33) when the protist community was examined as a whole (Fig. 5A). However, significant increases in latitudinal range were found for most individual protist clades, particularly dinoflagellates, Prasino-Clade-7, and Acantharea (R = −0.81, *P* < 0.0001; R = −0.89, *P* < 0.0001, and R = −0.75, *P* < 0.0001, respectively), supporting Rapoport’s rule. Notable exceptions from this overall trend were the diatoms (Bacillariophyceae), choanoflagellates, picomonads, and Mamiellophyceae, which all exhibited decreasing latitudinal range with increasing latitude (Fig. 5A).

ASV richness and Shannon Index of the protist community both strongly decreased with increasing latitude, indicating that marine protists also exhibit the LDG (Fig. 5B and C). The taxa with the highest correlation coefficients contributing to decreasing Shannon diversity towards the poles are Prymnesiophyceae, Dinophyceae, and Dictyochophyceae (R = 0.81, *P* < 0.0001; R = 0.74, *P* < 0.0001; and R = 0.73, *P* < 0.0001, respectively), while the clades with the strongest decrease in ASV richness with increasing latitude are Dinophyceae, Dictyochophyceae, and Chrysophyceae-Synurophyceae (R = 0.82, *P* < 0.0001; R = 0.73, *P* < 0.0001; and R = 0.68, *P* < 0.0001, respectively). However, these patterns are not shown by all groups, with several taxa exhibiting opposite trends. ASV richness increases towards the pole in diatoms, choanoflagellates, Filosa−Thecofilosea, and Mamiellophyceae, while the Shannon-Index additionally increases polewards in the Pelagophyceae but not in the choanoflagellates (Fig. 5B and C).

**Figure 6.**
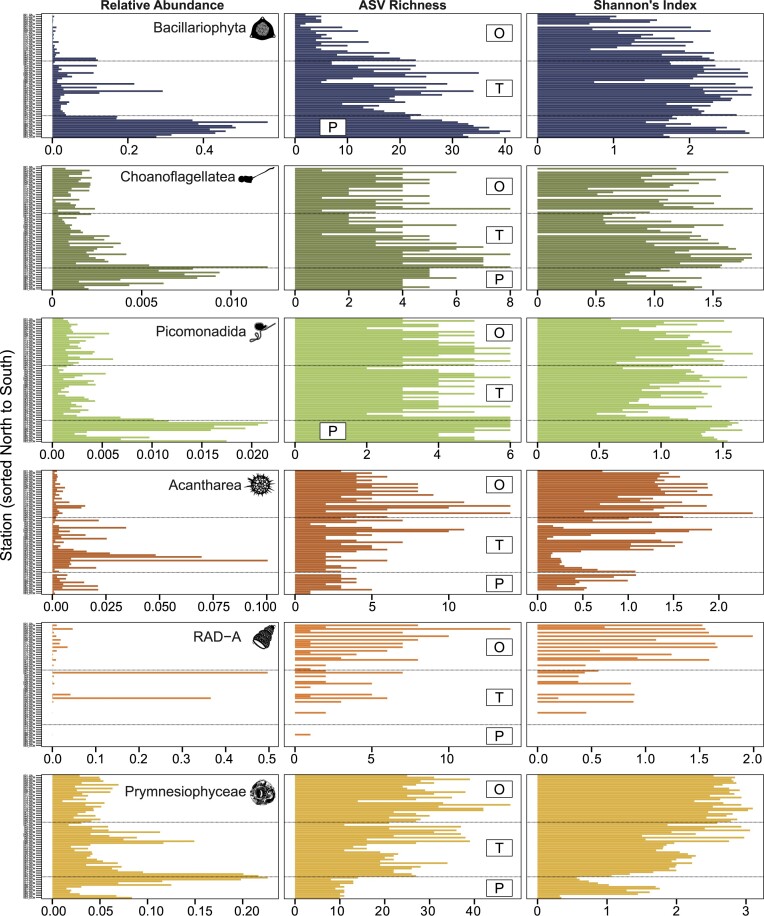
Clades with localized trends in abundance or diversity along the transect. Column 1: Relative Abundance; Column 2: ASV Richness; Column 3: Shannon’s Index. O: Oligotrophic Region, T: Temperate Region, P: Polar Region. Note the different x-axis scales of relative abundance (shown as proportion of total).

To understand why these groups do not appear to conform to the LDG, we examined how their diversity is influenced by relative abundance. Diatom ASVs have a very low relative abundance in the oligotrophic region, where ASV richness is also low (Fig. 6). The large increase in relative abundance in the polar region corresponds to a much greater ASV richness and an increase in Shannon’s Index. Choanoflagellate and picomonad ASVs also show a large increase in relative abundance within the polar region that corresponds to an increase in ASV richness. Spearman correlations between clade abundance and environmental variables (Supplementary Fig. S2) showed that diatom ASV abundance was correlated positively with several parameters, including increasing latitude, DIN, and silicate, as well as decreasing temperature and salinity. A similar trend, albeit not as pronounced, was evident in the choanoflagellates, the picomonads, and the Acantharea (Supplementary Fig. S2).

### A *Phaeocystis* bloom spanning the PF

As the SAZ represents an important transitionary boundary between coccolithophore communities with the Great Calcite Belt (GCB) and polar diatom communities (Oliver et al. 2023), we examined the distribution of the Prymnesiophyceae in more detail. Prymnesiophyceae ASVs are present throughout the transect, exhibiting a large increase in relative abundance at the transition from the temperate to the polar region, i.e. across the PF (Fig. 6). This was due to an increase in abundance of ASVs belonging to *Phaeocystis antartica*, the colonial haptophyte that forms large blooms in the SO (DiTullio et al. 2000) (Supplementary Fig. S3). Coccolithophore ASVs were present throughout the transect but generally at a low relative abundance, with Clade E and Coccolithales_X being found primarily in the oligotrophic region, *Gephyrocapsa* and *Braarudosphaera* predominantly in the temperate region, and *Pleurochrysis* in the polar region (Supplementary Fig. S3).

**Figure 7.**
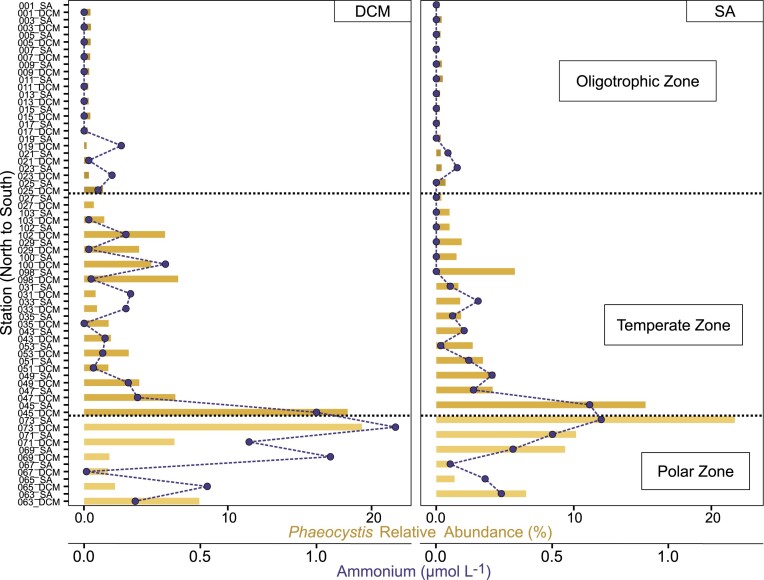
Relative abundance (%) of all ASVs assigned as Phaeocystis (bars) overlaid with ammonium concentrations (dots) at the DCM and surface (SA).

The position of the *Phaeocystis* bloom is interesting as many of the other ASVs that are abundant in the temperate region are absent from the polar region and do not span the PF (Fig. 3). To investigate this further, we examined whether abundance of *Phaeocystis* ASVs correlated to specific physical parameters. We found that *Phaeocystis* ASVs (ASV3 and ASV40) showed a strong positive correlation to ammonium concentrations (R = 0.71 and 0.63, *P* < 0.01, respectively) (Fig. 7). They also exhibited a positive, yet weaker, correlation with nitrate (R = 0.40 and 0.33, *P* < 0.01) and phosphate concentrations (R = 0.22 and 0.26, *P* < 0.01) (Fig. 7).

## Discussion

### Oceanographic features constrain protist communities

Microbial communities within the southern Pacific Ocean make a major contribution to oceanic CO_2_ drawdown (Frölicher et al. 2015) and underpin the rich diversity of life in these remote ecosystems. However, they remain poorly studied, limiting our ability to understand the environmental factors that determine diversity and function. Our results show that major oceanographic features, namely the Subtropical Front (STF) and the PF, play an important role in constraining these communities, with distinct communities found in the water masses defined by these features. Our high-resolution transect spanning >3000 km supports previous observations indicating that protist communities in the SO are likely to be strongly influenced by oceanographic features (Diez Moreno 2004, Raes et al. 2018). Strong differences have also been observed in the composition of archaeal and bacterial communities across these regions (Raes et al. 2018).

The oligotrophic region north of the STF (representing the STZ) was characterized by a high relative abundance of ASVs belonging to the alveolate groups Dinophyceae and Syndiniales. The strongest indicator for the oligotrophic region were the free-living flagellates Discoba. Additionally, we found that phototrophic taxa ASVs were relatively least abundant in the oligotrophic region, while ASVs from hetero- and mixotrophic taxa were more common. Non-plastidic protists therefore likely play a substantial role in oligotrophic low-chlorophyll regions. A key feature of this region was the distinct differences between protist communities at the surface and DCM, likely driven by greater stratification of oligotrophic environments, while more intense deep-mixing in polar environments causes greater homogeneity across populations (Chen et al. 2021).

The temperate region between the STF and PF (comprising of the SAZ and PFZ) also exhibited a high relative abundance of ASVs belonging to dinoflagellates and Syndiniales, supporting the findings of a previous study in the SAZ (Wolf et al. 2013). With an increase in macronutrients in the temperate region, clades from the photosynthetic green algae (Archaeplastida) were particularly successful. Our data show that several Archeaplastida clades, particularly the Prasino-Clade-7 and the Mamiellophyceae, are strong indicators for temperate low-silica, high-macronutrient environments, becoming virtually absent south of the PF. Additionally, we observed a substantial increase in relative abundance of pelagophytes at the southern end of the temperate region. Wolf et al. (2013) also observed a peak in pelagophyte abundance just north of the PF. Pelagophytes were strongly constrained by the PF and were largely absent from the AZ.

The PF is recognized as a prominent separator in community assemblage in the southern Pacific Ocean (Wolf et al. 2013). The strongest indicator clades in the macronutrient- and silica-rich polar region were diatoms, picomonads, and choanoflagellates. The sharp increase in the relative abundance of diatoms south of the PF is in close agreement with previous studies demonstrating elevated diatom abundance across most SO basins due to the increased availability of silicate (DeMaster 1981, Alvain et al. 2008). The diatom community was primarily made up of the chain-forming genera *Chaetoceros* and *Fragilariopsis*, and the centric diatom *Thalassiosira*, which have previously been identified as major contributors to diatom populations in the SO (Lasbleiz et al. 2016). Spring diatom blooms contribute a large portion of SO primary production (Smith and Asper 2001, Tremblay et al. 2002, Gilbertson et al. 2022), with large, heavily silicified diatoms, such as *Eucampia antarctica* and *F. kerguelensis*, responsible for the high levels of export in this region (Salter et al. 2007, Assmy et al. 2013). Frontal regions contribute to ecotypic differentiation and speciation in the diatom *F. kerguelensis* (Postel et al. 2020). The increase in choanoflagellate abundance in the polar region was primarily due to an increase in ASVs belong to the Stephanoecidae. This family of choanoflagellates possesses a basket-like lorica composed of siliceous ribs, aligning with the alleviation of Si limitation south of the PF.

### Phaeocystis blooms span the PF

The PF has a large impact on protist communities, with a strong shift towards dominance of siliceous protists such as diatoms and choanoflagellates in the silicate-rich polar waters. Low temperatures alongside strong competition for resources from the fast-growing diatoms, likely exclude many other protists from this region. However, not all taxa are constrained by this feature. Most notably, we identified a substantial bloom of *Phaeocystis antarctica* (which was also confirmed visually during sampling) that spanned the PF. Other studies of protist communities in this region have also identified a similar distribution for *Phaeocystis* blooms (Wolf et al. 2013, Sow et al. 2020). Large recurrent blooms of *P. antarctica* are common in high-nutrient, low chlorophyll (HNLC) regions of the SO, as well as in areas where silicate is exhausted or naturally limited (Schoemann et al. 2005, Salter et al. 2007). Within the silicate-rich AZ, *P. antarctica* is able to outcompete diatoms under certain conditions, such as low irradiances or at optimal temperatures, while diatoms perform better under low iron conditions (Schoemann et al. 2005). The relative dominance of *P. antarctica* or diatoms within the AZ is therefore largely controlled by seasonal differences in temperature and iron availability (Nissen and Vogt 2021, Smith and Trimborn 2023).

We did not measure Fe during our study, so we cannot determine whether it contributed to changes in the abundance of diatoms and *P. antarctica* within the polar region of the transect. However, we found that *P. antarctica* ASVs were associated with high concentrations of NH_4_^+^, which it might use as an N source (Wang et al. 2011, Smith and Trimborn 2023). *Phaeocystis* can exhibit higher rates of NH_4_^+^ uptake than diatoms (Tungaraza et al. 2003), although it seems unlikely that this has a strong influence on competition within the N-rich AZ. Instead, the elevated NH_4_^+^ associated with *P. antarctica* may reflect NH_4_^+^ released during the decomposition of senescent *Phaeocystis* populations (Tang et al. 2009, Delmont et al. 2014). While *P. antarctica* blooms can readily span the PF, these assemblages may still be strongly influenced by this feature, with a strong shift in the dominant *P. antarctica* ecotypes across the PF (Sow et al. 2020).

### LDG and Rapoport’s rule

We found that the overall protist community diversity (measured as Shannon’s H and ASV richness) adhered to the hypothesis of the LDG. In most clades, diversity was greatest in the oligotrophic northern region and decreased substantially with increasing latitude. This supports previous observations that eukaryotic richness was greatest in regions with lowest productivity (Raes et al. 2018). Similarly, Ibarbalz et al. (2019) demonstrated increased species richness of global plankton communities towards the equator using *Tara Oceans* data, which was linked to increased ocean temperatures. However, we observed that these relationships did not hold for all taxa when examined individually, with diatoms, choanoflagellates, pelagophytes, Filosa-Thecofilosea, and Mamiellophyceae increasing in diversity with increasing latitude. This result contrasts with analysis from the *Tara Oceans* dataset that shows a peak in diatom species richness at the equator (Ibarbalz et al. 2019). The differences are likely due to the very different spatial and temporal ranges of the respective datasets, including significant under-sampling of the mid to high latitudes in the southern hemisphere within *Tara Oceans* (Sow et al. 2020). Several studies have demonstrated that some marine organisms do not conform to the LDG, and instead exhibit a bimodal distribution with peaks in diversity at mid-latitudes (Chaudhary et al. 2016, Sow et al. 2020). Clearly, these contrasting findings can only be resolved with improved sampling resolution, particularly in the more remote areas of our oceans.

We found that latitudinal range was not significantly correlated to latitude when considering the entire protist community in the southern Pacific Ocean, supporting the conclusion of a previous study examining eukaryote diversity in the region during austral winter (Raes et al. 2018). Rapoport’s rule, which suggests that range sizes are largest near the pole and decrease towards the equator, therefore does not appear to apply for eukaryotic protists in the southern Pacific Ocean. By investigating latitudinal ranges of individual clades, we were able to reveal that Rapoport’s effect does in fact occur in several protist taxa. Most significantly, in dinoflagellates, Prasino-Clade-7, and acanthareans, while a strong opposite trend with narrowest ranges towards the pole was evident in diatoms, choanoflagellates, Picomonadida, and Mammiellophyceae. It has previously been suggested that Rapoport’s rule is a strictly local phenomenon (Rohde 1996, Gaston et al. 1998); however, our results indicate that clade-specific differences substantially impact community diversity trends.

### The GCB

The GCB is an annually reoccurring circumpolar band of elevated particulate inorganic carbon (PIC) between 40 and 60°S. However, little information exists about its extent and taxonomic composition in the Pacific sector of the SO (Balch et al. 2016, Nissen et al. 2018). Measurements of PIC during the cruise indicated elevated PIC in the SAZ, suggesting the emergence of a coccolithophore bloom (Oliver et al. 2023). While our metabarcoding data revealed the presence of coccolithophores throughout the transect, the relative abundance of coccolithophore ASVs remained low even in regions where PIC concentrations and counts of coccolithophore cells were highest (Oliver et al. 2023). It is likely that several coccolithophore species are under-represented or missing in our dataset entirely due to poor amplification of coccolithophore sequences with the universal eukaryotic 18S gene markers used in this study (Edvardsen et al. 2016). For example, the heavily calcified *Calcidiscus leptoporus*, which has been found to locally dominate coccolithophore communities in the Pacific SO, is absent from our dataset (Nissen et al. 2018). It should also be considered that poor amplification of coccolithophore sequences could also influence relative abundances of other taxa (ASVs). Future studies of coccolithophore populations in this region must therefore consider alternative methodologies (e.g. alternative primers or marker genes) to assess their contribution to the wider protist community. A combination of methodologies, such as using quantitative PCR or droplet digital PCR to target specific taxa alongside metabarcoding, has been used to increase the sensitivity of detection of under-represented taxa (Wood et al. 2019).

## Conclusion

Our comprehensive survey of marine microbial eukaryote communities in the remote southern Pacific Ocean demonstrates that these communities are strongly defined by major oceanographic features, such as the PF and STF, although some taxa are able to exploit and occupy these frontal environments. Our data also indicate that protist communities largely adhere to the LDG, with greatest diversity in lower latitudes, although some taxa (most notably the diatoms) exhibit an opposing trend. Future warming of the oceans is predicted to cause a poleward shift of many taxa (Ibarbalz et al. 2019), which could result in interactions with frontal regions. It is therefore vital that we gain a better understanding of the combined influence of oceanographic features and large-scale physical gradients such as temperature on current marine microbial communities, in order to predict their response to future environmental change.

## Supplementary Material

fiae137_Supplemental_File

## Data Availability

Raw sequencing reads are deposited in the Sequence Read Archive (SRA) (Accession PRJNA1044770). All data will be made available following publication.

## References

[bib1] Abbott MR, Richman JG, Letelier RM et al. The spring bloom in the Antarctic Polar Frontal Zone as observed from a mesoscale array of bio-optical sensors. Deep Sea Res Part II. 2000;47:3285–314.

[bib2] Alvain S, Moulin C, Dandonneau Y et al. Seasonal distribution and succession of dominant phytoplankton groups in the global ocean: a satellite view. Global Biogeochem Cycles. 2008;22:GB3001

[bib3] Amaral-Zettler LA, McCliment EA, Ducklow HW et al. A method for studying protistan diversity using massively parallel sequencing of V9 hypervariable regions of small-subunit ribosomal RNA genes. PLoS One. 2009;4:e6372. 19633714 10.1371/journal.pone.0006372 PMC2711349

[bib4] Amend AS, Oliver TA, Amaral-Zettler LA et al. Macroecological patterns of marine bacteria on a global scale. J Biogeogr. 2013;40:800–11.

[bib5] Anderson MJ . A new method for non-parametric multivariate analysis of variance. Austral Ecol. 2001;26:32–46.

[bib6] Assmy P, Smetacek V, Montresor M et al. Thick-shelled, grazer-protected diatoms decouple ocean carbon and silicon cycles in the iron-limited Antarctic Circumpolar Current. Proc Natl Acad Sci USA. 2013;110:20633–8. 24248337 10.1073/pnas.1309345110 PMC3870680

[bib7] Atlas EL, Hager SW, Gordon LI et al. A Practical Manual for Use of the Technicon AutoAnalyzer® in Seawater Nutrient Analyses Revised. Technical Report 215. Defense Technical Information Center, Fort Belvoir, VA: Oregon State University, Dept of Oceanography, 1971.

[bib8] Balch WM, Bates NR, Lam PJ et al. Factors regulating the Great Calcite Belt in the Southern Ocean and its biogeochemical significance. Global Biogeochem Cycles. 2016;30:1124–44.

[bib9] Barton AD, Dutkiewicz S, Flierl G et al. Patterns of diversity in marine phytoplankton. Science. 2010;327:1509–11. 20185684 10.1126/science.1184961

[bib10] Behrens E, Hogg AM, England MH et al. Seasonal and interannual variability of the subtropical front in the New Zealand region. JGR Oceans. 2021;126:e2020JC016412.

[bib11] Bradley IM, Pinto AJ, Guest JS. Design and evaluation of Illumina MiSeq-compatible, 18S rRNA gene-specific primers for improved characterization of mixed phototrophic communities. Appl Environ Microb. 2016;82:5878–91. 10.1128/AEM.01630-16 PMC5038042 27451454

[bib12] Burki F, Roger AJ, Brown MW et al. The new tree of eukaryotes. Trends Ecol Evol. 2020;35:43–55. 31606140 10.1016/j.tree.2019.08.008

[bib13] Callahan BJ, McMurdie PJ, Rosen MJ et al. DADA2: high-resolution sample inference from Illumina amplicon data. Nat Methods. 2016;13:581–3. 27214047 10.1038/nmeth.3869 PMC4927377

[bib14] Catlett D, Matson PG, Carlson CA et al. Evaluation of accuracy and precision in an amplicon sequencing workflow for marine protist communities. Limnol Oceanogr Methods. 2020;18:20–40.

[bib15] Chaudhary C, Saeedi H, Costello MJ. Bimodality of latitudinal gradients in marine species richness. Trends Ecol Evol. 2016;31:670–6. 27372733 10.1016/j.tree.2016.06.001

[bib16] Chen Z, Sun J, Gu T et al. Nutrient ratios driven by vertical stratification regulate phytoplankton community structure in the oligotrophic western Pacific Ocean. Ocean Sci. 2021;17:1775–89.

[bib17] Delmont TO, Hammar KM, Ducklow HW et al. *Phaeocystis antarctica* blooms strongly influence bacterial community structures in the Amundsen Sea polynya. Front Microbiol. 2014;5:646. 25566197 10.3389/fmicb.2014.00646 PMC4271704

[bib18] DeMaster DJ . The supply and accumulation of silica in the marine environment. Geochim Cosmochim Acta. 1981;45:1715–32.

[bib19] Deppeler SL, Davidson AT. Southern Ocean phytoplankton in a changing climate. Front Mar Sci. 2017;4:40.

[bib20] De Vargas C, Audic S, Henry N et al. Eukaryotic plankton diversity in the sunlit ocean. Science. 2015;348:1261605–1/11. 25999516 10.1126/science.1261605

[bib21] Diez Moreno B . Distribution of eukaryotic picoplankton assemblages across hydrographic fronts in the Southern Ocean, studied by denaturing gradient gel electrophoresis. Limonol Oceanogr. 2004;49:1022–34.

[bib22] DiTullio GR, Grebmeier JM, Arrigo KR et al. Rapid and early export of *Phaeocystis antarctica* blooms in the Ross Sea, Antarctica. Nature. 2000;404:595–8. 10766240 10.1038/35007061

[bib23] Doblin MA, Petrou KL, Shelly K et al. Diel variation of chlorophyll-a fluorescence, phytoplankton pigments and productivity in the Subantarctic and polar Front Zones south of Tasmania, Australia. Deep Sea Res Part II. 2011;58:2189–99.

[bib24] Dufrêne M, Legendre P. Species assemblages and indicator species: the need for a flexible asymmetrical approach. Ecol Monogr. 1997;67:345–66.

[bib25] Edvardsen B, Egge ES, Vaulot D. Diversity and distribution of haptophytes revealed by environmental sequencing and metabarcoding—a review. Perspect Phycol. 2016;3:77–91.

[bib26] Flegontova O, Flegontov P, Malviya S et al. Extreme diversity of diplonemid eukaryotes in the ocean. Curr Biol. 2016;26:3060–5. 27875689 10.1016/j.cub.2016.09.031

[bib27] Frölicher TL, Sarmiento JL, Paynter DJ et al. Dominance of the Southern Ocean in anthropogenic carbon and heat uptake in CMIP5 models. J Clim. 2015;28:862–86.

[bib28] Gaston KJ, Blackburn TM, Spicer JI. Rapoport's rule: time for an epitaph?. Trends Ecol Evol. 1998;13:70–4. 21238203 10.1016/s0169-5347(97)01236-6

[bib29] Gilbertson R, Langan E, Mock T. Diatoms and their microbiomes in complex and changing polar oceans. Front Microbiol. 2022;13:786764. 35401494 10.3389/fmicb.2022.786764 PMC8991070

[bib30] Gong W, Marchetti A. Estimation of 18S gene copy number in marine eukaryotic plankton using a next-generation sequencing approach. Front Mar Sci. 2019;6:219

[bib31] Gordon LI, Jennings JC Jr, Ross AA et al. A suggested protocol for continuous flow automated analysis of seawater nutrients (phosphate, nitrate, nitrite and silicic acid) in the WOCE Hydrographic Program and the Joint Global Ocean Fluxes Study. WOCE Hydrogr Progr Off Methods Man WHPO. 1993:1–52.

[bib32] Gran-Stadniczeñko S, Egge E, Hostyeva V et al. Protist diversity and seasonal dynamics in Skagerrak Plankton communities as revealed by metabarcoding and microscopy. J Eukaryotic Microbiology. 2019;66:494–513. 30414334 10.1111/jeu.12700 PMC6587730

[bib33] Guillou L, Bachar D, Audic S et al. The Protist Ribosomal Reference database (PR2): a catalog of unicellular eukaryote small sub-unit rRNA sequences with curated taxonomy. Nucleic Acids Res. 2012;41:D597–604. 23193267 10.1093/nar/gks1160 PMC3531120

[bib34] Gutiérrez-Rodríguez A, dos Santos AL, Safi K et al. Planktonic protist diversity across contrasting Subtropical and Subantarctic waters of the southwest Pacific. Prog Oceanogr. 2022;206:102809.

[bib35] Hager SW, Atlas EL, Gordon LI et al. A comparison at sea of manual and autoanalyzer analyses of phosphate, nitrate, and silicate. Limnol Oceanogr. 1972;17:931–7.

[bib36] Hiscock MR, Marra J, Jr S et al. Primary productivity and its regulation in the Pacific Sector of the Southern Ocean. Deep Sea Res Part II. 2003;50:533–58.

[bib37] Honjo S . Particle export and the biological pump in the Southern Ocean. Antartic Sci. 2004;16:501–16.

[bib38] Hutchins DA, Fu F. Microorganisms and ocean global change. Nat Microbiol. 2017;2:1–11. 10.1038/nmicrobiol.2017.58 28540925

[bib39] Ibarbalz FM, Henry N, Brandão MC et al. Global trends in marine plankton diversity across kingdoms of life. Cell. 2019;179:1084–1097.e21. 31730851 10.1016/j.cell.2019.10.008 PMC6912166

[bib40] Jablonski D, Huang S, Roy K et al. Shaping the latitudinal diversity gradient: new perspectives from a synthesis of paleobiology and biogeography. Am Nat. 2017;189:1–12. 28035884 10.1086/689739

[bib41] Kim YS, Orsi AH. On the variability of Antarctic Circumpolar Current fronts inferred from 1992–2011 altimetry. J Phys Oceanogr. 2014;44:3054–71.

[bib42] Landschützer P, Gruber N, Haumann FA et al. The reinvigoration of the Southern Ocean carbon sink. Science. 2015;349:1221–4. 26359401 10.1126/science.aab2620

[bib43] Lasbleiz M, Leblanc K, Armand LK et al. Composition of diatom communities and their contribution to plankton biomass in the naturally iron-fertilized region of Kerguelen in the Southern Ocean. FEMS Microbiol Ecol. 2016;92:fiw171. 27515734 10.1093/femsec/fiw171

[bib44] Martin JL, Santi I, Pitta P et al. Towards quantitative metabarcoding of eukaryotic plankton: an approach to improve 18S rRNA gene copy number bias. MBMG. 2022;6:e85794.

[bib45] Martin M . Cutadapt removes adapter sequences from high-throughput sequencing reads. EMBnet J. 2011;17:10–2.

[bib46] McMurdie PJ, Holmes S. phyloseq: an R package for reproducible interactive analysis and graphics of microbiome census data. PLoS One. 2013;8:e61217. 23630581 10.1371/journal.pone.0061217 PMC3632530

[bib47] Mittelbach GG, Schemske DW, Cornell HV et al. Evolution and the latitudinal diversity gradient: speciation, extinction and biogeography. Ecol Lett. 2007;10:315–31. 17355570 10.1111/j.1461-0248.2007.01020.x

[bib48] Nissen C, Vogt M, Münnich M et al. Factors controlling coccolithophore biogeography in the Southern Ocean. Biogeosciences. 2018;15:6997–7024.

[bib49] Nissen C, Vogt M. Factors controlling the competition between phaeocystis and diatoms in the Southern Ocean and implications for carbon export fluxes. Biogeosciences. 2021;18:251–83.

[bib50] Oliver H, McGillicuddy DJ Jr, Krumhardt KM et al. Environmental drivers of coccolithophore growth in the Pacific sector of the Southern Ocean. Global Biogeochem Cycles. 2023;37:e2023GB007751.

[bib51] Orsi AH, Whitworth T, Nowlin WD. On the meridional extent and fronts of the Antarctic Circumpolar Current. Deep Sea Res Part I. 1995;42:641–73.

[bib52] Pawlowski J, Lejzerowicz F, Apotheloz-Perret-Gentil L et al. Protist metabarcoding and environmental biomonitoring: time for change. Eur J Protistol. 2016;55:12–25. 27004417 10.1016/j.ejop.2016.02.003

[bib53] Postel U, Glemser B, Salazar Alekseyeva K et al. Adaptive divergence across Southern Ocean gradients in the pelagic diatom *Fragilariopsis kerguelensis*. Mol Ecol. 2020;29:4913–24. 32672394 10.1111/mec.15554

[bib54] Raes EJ, Bodrossy L, Van De Kamp J et al. Oceanographic boundaries constrain microbial diversity gradients in the south pacific ocean. Proc Natl Acad Sci USA. 2018;115:E8266–75. 30108147 10.1073/pnas.1719335115 PMC6126737

[bib55] Richardson AJ, Schoeman DS. Climate impact on plankton ecosystems in the Northeast Atlantic. Science. 2004;305:1609–12. 15361622 10.1126/science.1100958

[bib56] Rigual-Hernández AS, Trull TW, Bray SG et al. Seasonal dynamics in diatom and particulate export fluxes to the deep sea in the Australian sector of the southern Antarctic Zone. J Mar Syst. 2015;142:62–74.

[bib57] Rohde K . Rapoport's rule is a local phenomenon and cannot explain latitudinal gradients in species diversity. Biodivers Lett. 1996;3:10–3.

[bib58] Salter I, Lampitt RS, Sanders R et al. Estimating carbon, silica and diatom export from a naturally fertilised phytoplankton bloom in the Southern Ocean using PELAGRA: a novel drifting sediment trap. Deep Sea Res Part II. 2007;54:2233–59.

[bib59] Santoferrara L, Burki F, Filker S et al. Perspectives from ten years of protist studies by high-throughput metabarcoding. J Eukaryot Microbiol. 2020;67:612–22. 32498124 10.1111/jeu.12813

[bib60] Schoemann V, Becquevort S, Stefels J et al. Phaeocystis blooms in the global ocean and their controlling mechanisms: a review. J Sea Res. 2005;53:43–66.

[bib61] Smith WO Jr, Asper VL. The influence of phytoplankton assemblage composition on biogeochemical characteristics and cycles in the southern Ross Sea, Antarctica. Deep Sea Res Part I. 2001;48:137–61.

[bib62] Smith WO Jr, Trimborn S. *Phaeocystis*: a Global Enigma. Annu Rev Mar Sci. 2023;16:417–41. 10.1146/annurev-marine-022223-025031 37647611

[bib63] Sow SLS, Trull TW, Bodrossy L. Oceanographic fronts shape *Phaeocystis* assemblages: a high-resolution 18S rRNA gene survey from the ice-edge to the equator of the South Pacific. Front Microbiol. 2020;11:1847. 32849444 10.3389/fmicb.2020.01847 PMC7424020

[bib64] Stern R, Kraberg A, Bresnan E et al. Molecular analyses of protists in long-term observation programmes—Current status and future perspectives. J Plankton Res. 2018;40:519–36.

[bib65] Stevens GC . the elevational gradient in altitudinal range: an extension of Rapoport's latitudinal rule to altitude. Am Nat. 1992;140:893–911. 19426029 10.1086/285447

[bib66] Sul WJ, Oliver TA, Ducklow HW et al. Marine bacteria exhibit a bipolar distribution. Proc Natl Acad Sci USA. 2013;110:2342–7. 23324742 10.1073/pnas.1212424110 PMC3568360

[bib67] Tang KW, Jr S, W. O et al. Survival and recovery of *Phaeocystis antarctica* (Prymnesiophyceae) from prolonged darkness and freezing. Proc Biol Sci. 2009;276:81–90. 18765338 10.1098/rspb.2008.0598 PMC2614241

[bib68] Tremblay JE, Lucas MI, Kattner G et al. Significance of the Polar Frontal Zone for large-sized diatoms and new production during summer in the Atlantic sector of the Southern Ocean. Deep Sea Res Part II. 2002;49:3793–811.

[bib69] Trull TW, Bray SG, Manganini SJ et al. Moored sediment trap measurements of carbon export in the Subantarctic and Polar Frontal Zones of the Southern Ocean, south of Australia. J Geophys Res. 2001;106:31489–509.

[bib70] Tungaraza C, Rousseau V, Brion N et al. Contrasting nitrogen uptake by diatom and phaeocystis-dominated phytoplankton assemblages in the North Sea. J Exp Mar Biol Ecol. 2003;292:19–41.

[bib71] Von Dassow P, John U, Ogata H et al. Life-cycle modification in open oceans accounts for genome variability in a cosmopolitan phytoplankton. ISME J. 2015;9:1365–77. 25461969 10.1038/ismej.2014.221 PMC4438323

[bib72] Wang X, Wang Y, Smith WO Jr. The role of nitrogen on the growth and colony development of phaeocystis globosa (Prymnesiophyceae). Eur J Phycol. 2011;46:305–14.

[bib73] Wickham H, Chang W, Wickham MH. Package ‘ggplot2’. *Creat. Elegant data vis. Using Gramm*. Graph Version. 2016;2:1–189.

[bib74] Wolf C, Frickenhaus S, Kilias ES et al. Protist community composition in the Pacific sector of the Southern Ocean during austral summer 2010. Polar Biol. 2014;37:375–89.

[bib75] Wood SA, Pochon X, Laroche O et al. A comparison of droplet digital polymerase chain reaction (PCR), quantitative PCR and metabarcoding for species-specific detection in environmental DNA. Mol Ecol Resour. 2019;19:1407–19. 31293089 10.1111/1755-0998.13055

[bib76] Worden AZ, Follows MJ, Giovannoni SJ et al. Rethinking the marine carbon cycle: factoring in the multifarious lifestyles of microbes. Science. 2015;347:1257594. 25678667 10.1126/science.1257594

