# Supplementary material for: Latitudinal gradients and ocean fronts strongly influence protist communities in the southern Pacific Ocean
Source: FEMS Microbiol Ecol. 2024 Nov 11;100(12):fiae137. doi: 10.1093/femsec/fiae137 (PMC11653569; doi:10.1093/femsec/fiae137)
Supplement: fiae137_Supplemental_File [file fiae137_supplemental_file.docx]

Supplementary Figures


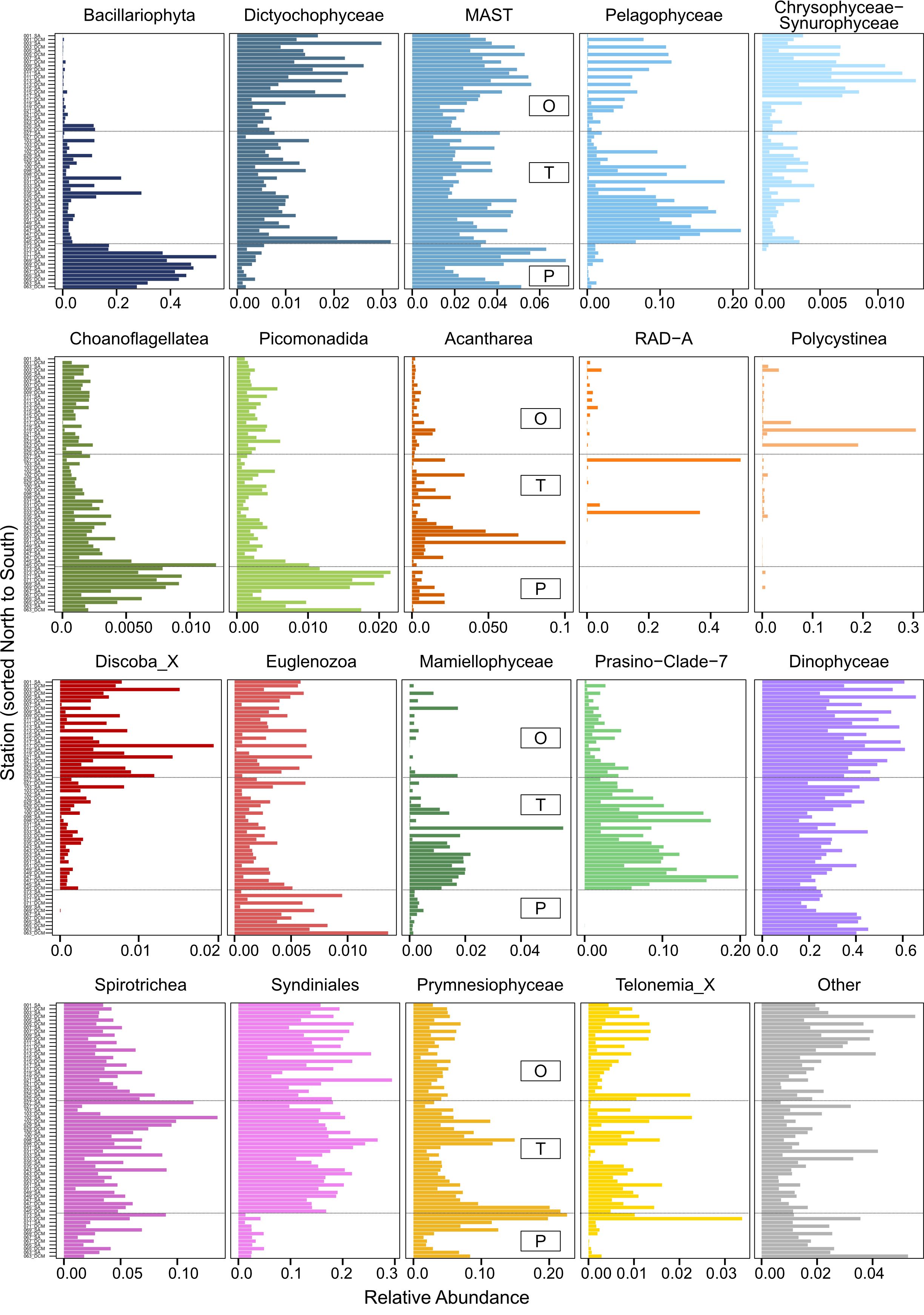


**Supplementary Figure 1 Relative abundances of 20 most abundant Phyla along the transect.** Stations (y-axis) are sorted by Latitude from North to South. Lines indicate the boundaries of the different environmental regions (O: Oligotrophic Region, T: Temperate Region, P: Polar Region). Note the different scales along the x-axis. Relative abundance is shown as proportion of total.

*
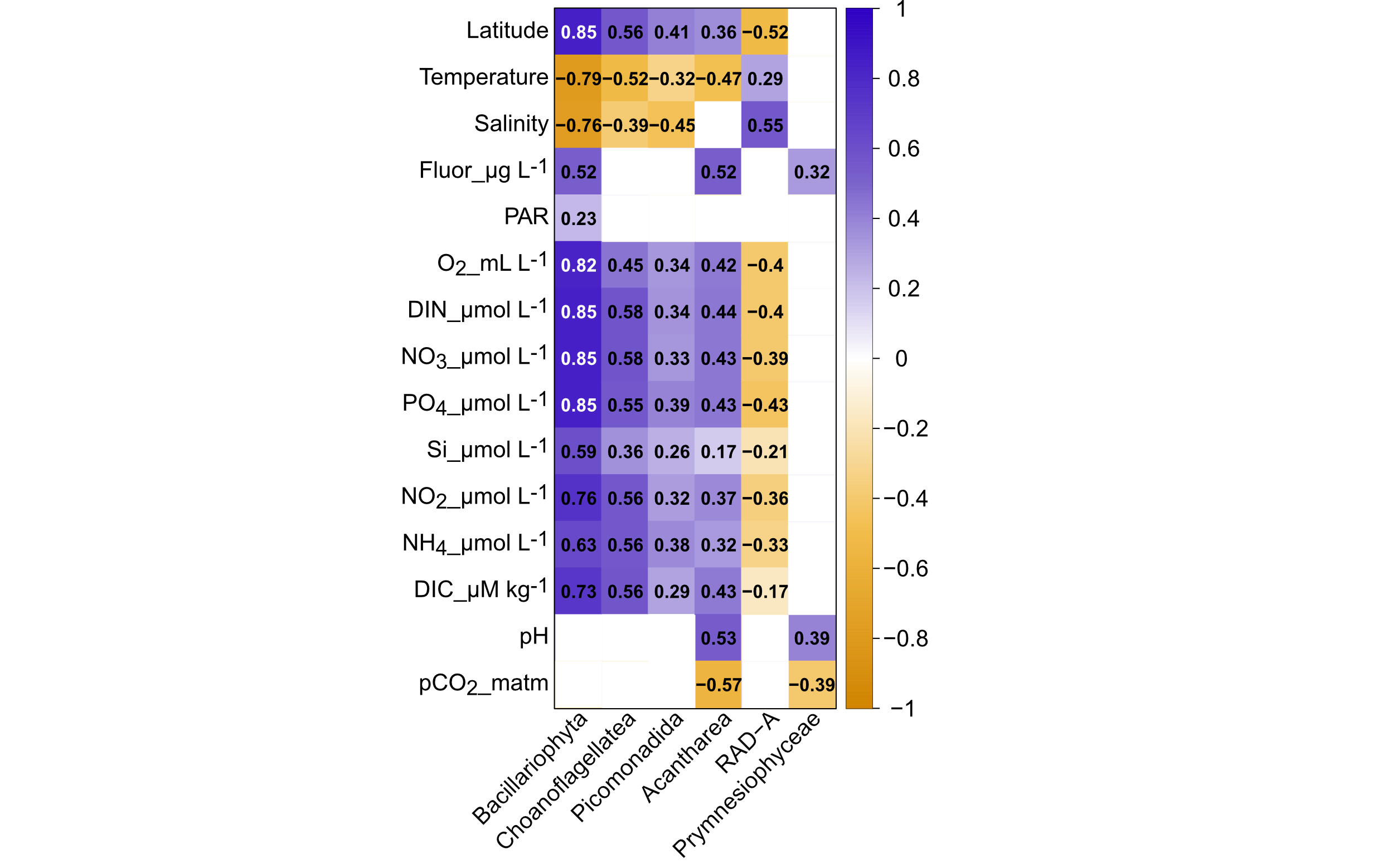
*

**Supplementary Figure 2 Environmental correlations (Spearman) of clades with localized abundance trends.** Spearman correlations of phyla with abundance trends along the transect (Bacillariophyta, Choanoflagellatea, Picomonadida, Acantharea, RAD-A, Prymnesiophyceae) and environmental variables. Only correlations with p < 0.01 are shown. Number inside the box represents correlation coefficient R.


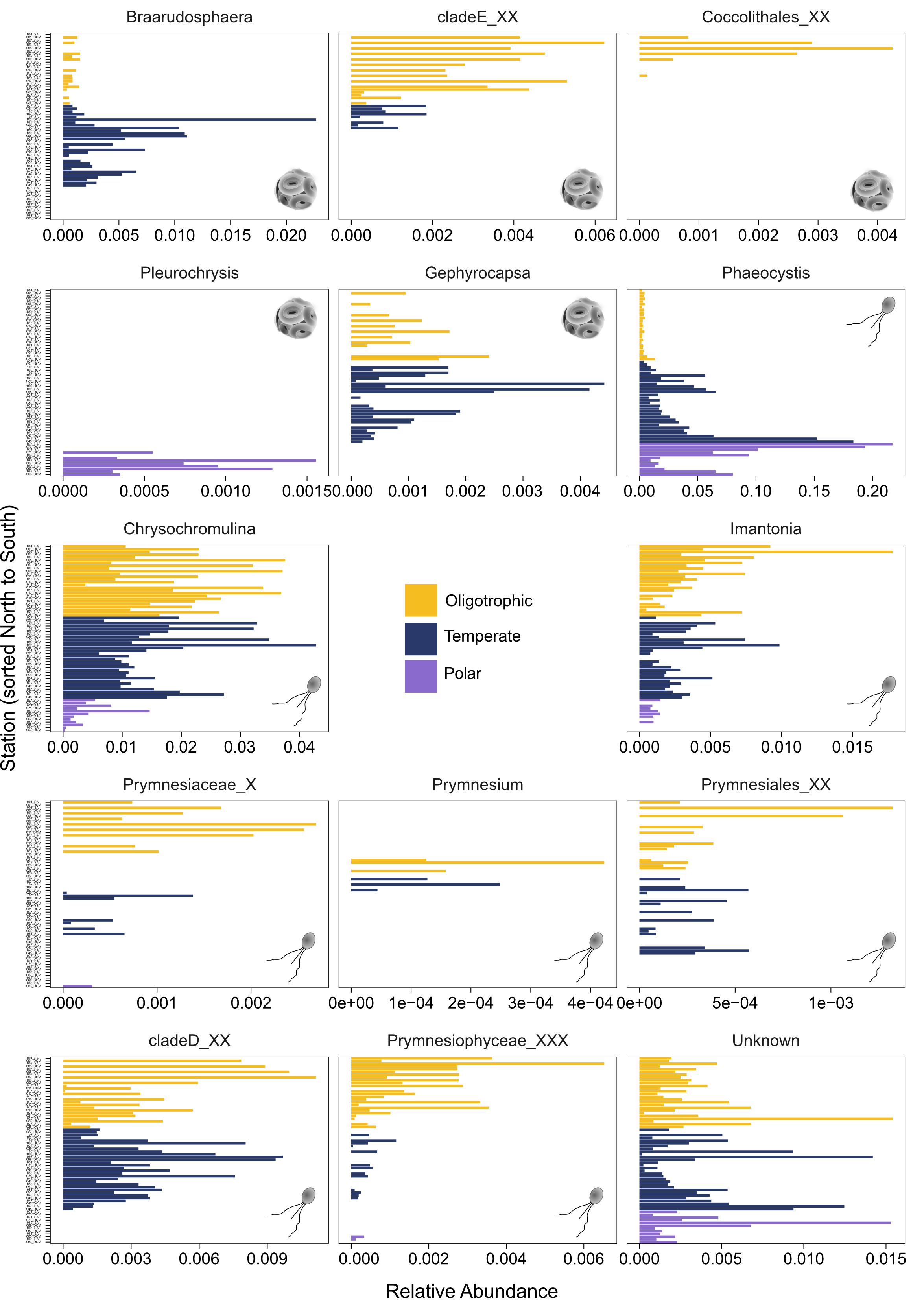


**Supplementary Figure 3 Relative abundances of all genera within the Prymnesiophyceae clade.** Calcifying Prymnesiophyceae are indicated by the coccolithophore shape, and non-calcifying clades by the flagellated shape. Relative abundance is shown as proportion of total.

Supplementary Tables

**Supplementary Table 1** PCR primers used in this study.

| Name | Direction | Sequence (5’-3’) | Product Size (bp) |
| --- | --- | --- | --- |
| Illumina_Euk_1391f | F | GTACACACCGCCCGTC | 160 |
| Illumina_EukBr | R | TGATCCTTCTGCAGGTTCACCTAC |  |

**Supplementary Table 2** The 24 most abundance Phyla along the transect and their respective trophic strategies.

| Taxonomic Group 1 | Taxonomic Group 2 | Trophic Strategy |
| --- | --- | --- |
| Stramenopila | Bacillariophyta | Phototrophic |
|  | Dictyochophyceae | Phototrophic |
|  | MAST | Heterotrophic |
|  | Pelagophyceae | Phototrophic |
|  | Chrysophyceae-Synurophyceae | Mixotrophic |
| Rhizaria | Acantharea | Heterotrophic |
|  | RAD-A | Heterotrophic |
|  | RAD-B | Heterotrophic |
|  | Polycystinea | Heterotrophic |
|  | Filosa-Thecofilosea | Heterotrophic |
| Hacrobia | Katablepharidaceae | Heterotrophic |
|  | Prymnesiophyceae | Phototrophic |
|  | Telonemia_X | Heterotrophic |
|  | Cryptophyceae | Phototrophic |
| Excavata | Discoba_X | Mixotrophic |
|  | Euglenozoa | Mixotrophic |
| Archaeplastida | Mamiellophyceae | Phototrophic |
|  | Prasino-Clade-7 | Phototrophic |
| Alveolata | Dinophyceae | Mixotrophic |
|  | Oligohymenophorea | Heterotrophic |
|  | Spirotrichea | Heterotrophic |
|  | Syndiniales | Heterotrophic |
| Opisthokonta | Choanoflagellatea | Heterotrophic |
| Picozoa | Picomonadida | Heterotrophic |

**Supplementary Table 3** Mean, Standard deviation, Minimum, and Maximum relative abundances for the Top 20 most abundant Phyla along the transect.

| Taxonomic Group | Supergroup | Mean | Stand dev | Min | Max |
| --- | --- | --- | --- | --- | --- |
| Dinophyceae | Alveolata | 35.06% | 12.49% | 15.68% | 65.60% |
| Syndiniales | Alveolata | 14.27% | 6.87% | 1.26% | 29.46% |
| Bacillariophyta | Stramenopila | 9.52% | 14.96% | 0.03% | 57.03% |
| Prymnesiophyceae | Hacrobia | 6.01% | 4.70% | 1.06% | 22.63% |
| Pelagophyceae | Stramenopila | 5.14% | 5.75% | 0.03% | 21.08% |
| Spirotrichea | Alveolata | 4.72% | 2.51% | 1.03% | 13.55% |
| Prasino-Clade-7 | Archaeplastida | 4.40% | 4.70% | 0.00% | 19.76% |
| MAST | Stramenopila | 3.24% | 1.36% | 1.00% | 7.23% |
| RAD-A | Rhizaria | 1.59% | 7.43% | 0.00% | 49.69% |
| Polycystinea | Rhizaria | 1.04% | 4.35% | 0.00% | 30.49% |
| Acantharea | Rhizaria | 0.95% | 1.62% | 0.00% | 10.04% |
| Dictyochophyceae | Stramenopila | 0.95% | 0.69% | 0.10% | 3.17% |
| Telonemia_X | Hacrobia | 0.64% | 0.63% | 0.00% | 3.38% |
| Mamiellophyceae | Archaeplastida | 0.61% | 0.92% | 0.00% | 5.52% |
| Picomonadida | Picozoa | 0.44% | 0.50% | 0.05% | 2.16% |
| Euglenozoa | Excavata | 0.33% | 0.25% | 0.02% | 1.36% |
| Discoba_X | Excavata | 0.32% | 0.41% | 0.00% | 1.95% |
| Chrysophyceae-Synurophyceae | Stramenopila | 0.26% | 0.29% | 0.00% | 1.33% |
| Choanoflagellatea | Opisthokonta | 0.25% | 0.24% | 0.00% | 1.21% |
| Unknown |  | 8.38% | 6.25% | 0.75% | 37.54% |
| Other |  | 1.88% | 1.12% | 0.60% | 5.53% |
